# Supplementary material for: Resolving the full spectrum of human genome variation using Linked-Reads
Source: Genome Res. 2019 Apr;29(4):635–45. doi: 10.1101/gr.234443.118 (PMC6442396; doi:10.1101/gr.234443.118)
Supplement: Supplemental Material [file supp_gr.234443.118_Supplemental_longranger-2.2.zip › longranger-2.2.tar.bz2!/lr2.2-public/lib/stan/cmdstan/stan/doc/api/html/math_2functions_2modified__bessel__first__kind_8hpp_source.html]

Stan: src/stan/math/functions/modified\_bessel\_first\_kind.hpp Source File


|  |  |
| --- | --- |
|  | Stan  2.5.0  probability, sampling & optimization |


- Main Page
- Related Pages
- Namespaces
- Classes
- Files

- File List
- File Members

All Classes Namespaces Files Functions Variables Typedefs Enumerations Enumerator Friends Macros Pages

- src
- stan
- math
- functions

modified\_bessel\_first\_kind.hpp

Go to the documentation of this file.

1 #ifndef STAN\_\_MATH\_\_FUNCTIONS\_\_MODIFIED\_BESSEL\_FIRST\_KIND\_HPP

2 #define STAN\_\_MATH\_\_FUNCTIONS\_\_MODIFIED\_BESSEL\_FIRST\_KIND\_HPP

3

4 #include <boost/math/special\_functions/bessel.hpp>

5 #include <stan/math/error\_handling/check\_not\_nan.hpp>

6

7 namespace stan {

8  namespace math {

9

37  template<typename T2>

38  inline T2

39  modified\_bessel\_first\_kind(const int v, const T2 z) {

40  check\_not\_nan("modified\_bessel\_first\_kind(%1%)", z, "z", static\_cast<double\*>(0));

41

42  return boost::math::cyl\_bessel\_i(v,z);

43  }

44

45  }

46 }

47

48 #endif

check\_not\_nan.hpp

stan::math::check\_not\_nan

bool check\_not\_nan(const char \*function, const T\_y &y, const char \*name, T\_result \*result)

Checks if the variable y is nan.

**Definition:** check\_not\_nan.hpp:56

stan::math::modified\_bessel\_first\_kind

T2 modified\_bessel\_first\_kind(const int v, const T2 z)

**Definition:** modified\_bessel\_first\_kind.hpp:39


---

[ Stan Home Page ]

*© 2011–2014,
Stan Development Team.*
